# Unraveling the mysteries of volatile cycling and habitability on Earth and beyond

**DOI:** 10.1093/nsr/nwae214

**Published:** 2024-06-20

**Authors:** Yi-Gang Xu, Ri-Xiang Zhu, Min-Han Dai

**Affiliations:** State Key Laboratory of Isotope Geochemistry, Guangzhou Institute of Geochemistry, Chinese Academy of Sciences, China; State Key Laboratory of Lithosphere Evolution, Institute of Geology and Geophysics, Chinese Academy of Sciences, China; State Key Laboratory of Marine Environmental Science, Xiamen University, China

The origin and cycling of volatiles, including water, carbon dioxide, and nitrogen, are fundamental to the very existence of habitable planets. These volatile compounds, often taken for granted, are the lifeblood of planetary systems, influencing almost everything from atmospheric composition and climate regulation to deep Earth processes and the emergence of life. Understanding the intricate processes that govern the distribution and evolution of these volatiles is paramount to unraveling the mysteries of planetary habitability and the potential for life beyond Earth. This Special Topic, featuring four perspective papers, three comprehensive reviews, and an insightful interview, delves into the origin and cycling of volatiles in habitable planets, offering a multifaceted exploration of this captivating field.

The quest to understand volatile elements naturally begins with our own celestial neighbor, the Moon. Hui *et al.* tackle the intriguing question of the origin of water on the Moon [1]. Their work sheds light on the mechanisms of volatile delivery and retention on airless bodies, providing valuable insights into the broader processes shaping volatile distribution in the early solar system. Shifting our focus to Mars, Hu *et al.* delve into the evidence for water and other volatiles on the Red Planet [2]. Their perspective explores the tantalizing possibility of past or present Martian habitability, highlighting the crucial role of volatiles in shaping the planet's history and potential for life.

Moving beyond individual planetary bodies, this Special Topic also grapples with the large-scale processes that govern volatile cycling. Wang and Xu provide a fascinating glimpse into the deep Earth, exploring the behavior of volatiles in the mantle transition zone [3]. Their work demonstrates the profound influence of these deep-seated volatiles on mantle dynamics, particularly their role in driving big mantle wedge systems and ultimately influencing surface processes. Lu *et al.* take a broader perspective, examining the coevolution of atmospheric oxygen and carbon dioxide levels throughout Earth's history [4]. Their perspective underscores the intricate interplay between these two critical volatiles and their combined impact on ocean chemistry and habitability, offering new insights into the long-term evolution of Earth's biosphere.

Complementing these perspectives, three comprehensive reviews elucidate the intricate mechanisms of volatile cycling and storage within Earth's interior. Foley *et al.* provide a detailed examination of carbon storage and release in the continental mantle, highlighting the significant impact of local variations in pressure, temperature, and geological processes on carbon cycling [5]. Zhang *et al.* expand the scope to a global scale, exploring deep carbon recycling within the framework of plate tectonics [6]. Their review underscores the crucial role of plate tectonics in regulating Earth's long-term carbon cycle, with implications for understanding both past and future climate evolution. Finally, Li provides a comprehensive overview of the origin and evolution of Earth's nitrogen [7]. This work traces the journey of this essential element from its primordial sources to our current understanding of its budget and isotope composition of different reservoirs, highlighting the complex interplay between geological and biological processes in affecting nitrogen evolution.

In the engaging interview with Professor Charles H. Langmuir, he shares his expertise and thoughts on the key factors that contribute to planetary habitability, the role of volatiles in shaping Earth-like environments, and the challenges and opportunities in the search for habitable worlds beyond our solar system [8].

The articles presented in this Special Topic collectively underscore the complex nature of volatiles in habitable planets. From their initial origins and delivery mechanisms to their complex cycling and storage within planetary interiors, volatiles are essential actors in the grand narrative of planetary evolution. By bringing together diverse perspectives from various disciplines and focusing on a range of planetary bodies, this collection of articles offers a comprehensive overview of the current state of knowledge and the exciting research frontiers in this captivating field. The insights gleaned from studying the origin and cycling of volatiles in habitable planets not only deepen our understanding of Earth's unique evolutionary trajectory but also serve as crucial guideposts in our ongoing search for potentially habitable worlds elsewhere in the vast expanse of the cosmos.

We would like to extend our sincere gratitude to all the authors, reviewers, and editorial staff who have contributed their time, expertise, and dedication to the development and completion of this Special Topic.
